# Idiopathic full thickness macular hole in a 10-year-old girl

**DOI:** 10.1186/s40942-018-0128-9

**Published:** 2018-07-11

**Authors:** Li-Anne S. Lim, Guillermo Fernandez-Sanz, Steven Levasseur, John R. Grigg, Alex P. Hunyor

**Affiliations:** 1 Sydney Hospital and Sydney Eye Hospital, Macquarie Street, Sydney, NSW Australia; 2 Discipline of Ophthalmology, Sydney Eye Hospital Campus, University of Sydney, Macquarie Street, Sydney, NSW Australia

**Keywords:** Macular hole, Children

## Abstract

**Background:**

Macular holes in children are generally associated with trauma.

**Case presentation:**

We report the first case of an idiopathic full thickness macular hole in a 10-year-old girl. 23-gauge transconjunctival pars plana vitrectomy, induction of a posterior vitreous detachment, ILM blue-assisted internal limiting membrane peel, fluid–air exchange and air-26% sulfur hexafluoride (SF6) exchange was performed with subsequent macular hole closure.

**Conclusion:**

This is the first reported case of an idiopathic full thickness macular hole in a child. Treatment with pars plana vitrectomy with peeling of the ILM resulted in significant anatomic and functional improvement.

## Background

A macular hole is a full thickness defect in the neural retina at the fovea. It is thought to occur as a result of pathological changes at the foveal vitreoretinal interface [1]. Idiopathic macular holes most commonly occur in adults in the 6th to 7th decade [2]. Macular holes in children are rare and are generally associated with trauma [3]. We present a case of an idiopathic full thickness macular hole (FTMH) in a child.

## Case presentation

A 10-year-old girl presented with reduced vision in the right eye. The vision had deteriorated from 20/17 1 year previously, to 20/60. She was otherwise well, with no history of trauma or inflammation of either eye and no other significant medical or drug history. Her grandfather, and grandfather’s brother had a history of retinal detachment.

**Fig. 1:**
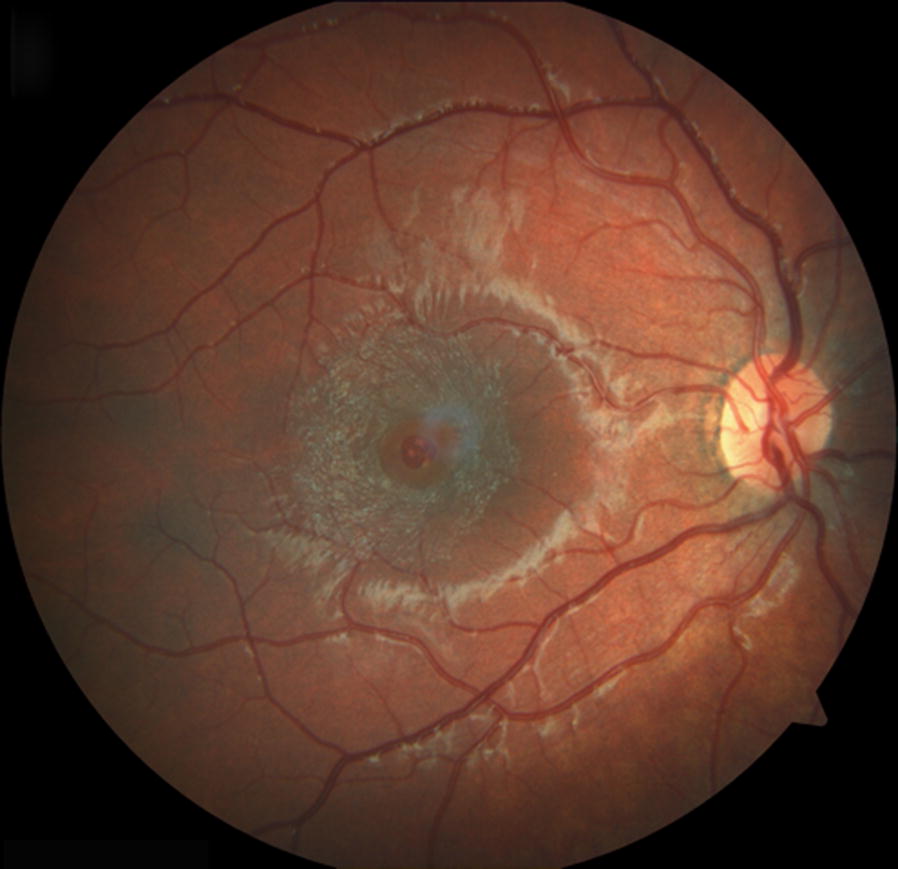
Colour fundus photograph of the right eye shows a full thickness macular hole

**Fig. 2:**
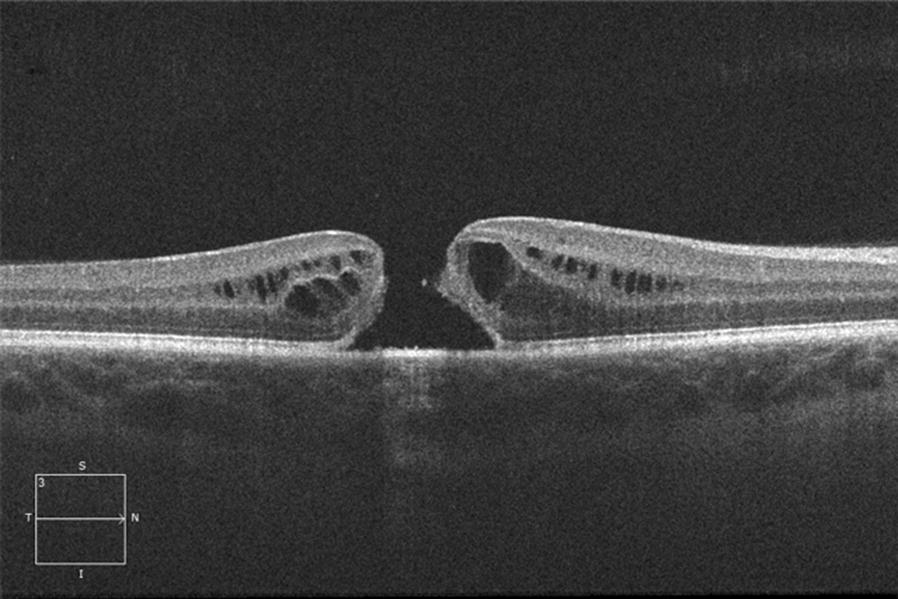
Horizontal high definition spectral domain OCT of the right eye shows a full thickness macular hole

Visual acuity (VA) was 20/60 in the right eye and 20/20 in the left eye. Ocular examination was unremarkable except for the presence of a FTMH in the right eye (Fig. 1). There was no evidence of trauma, inflammation or signs of retinal dystrophy. Optical coherence tomography (OCT) showed a 365 μm FTMH with no vitreomacular traction or posterior vitreous detachment (Fig. 2).

**Fig. 3:**
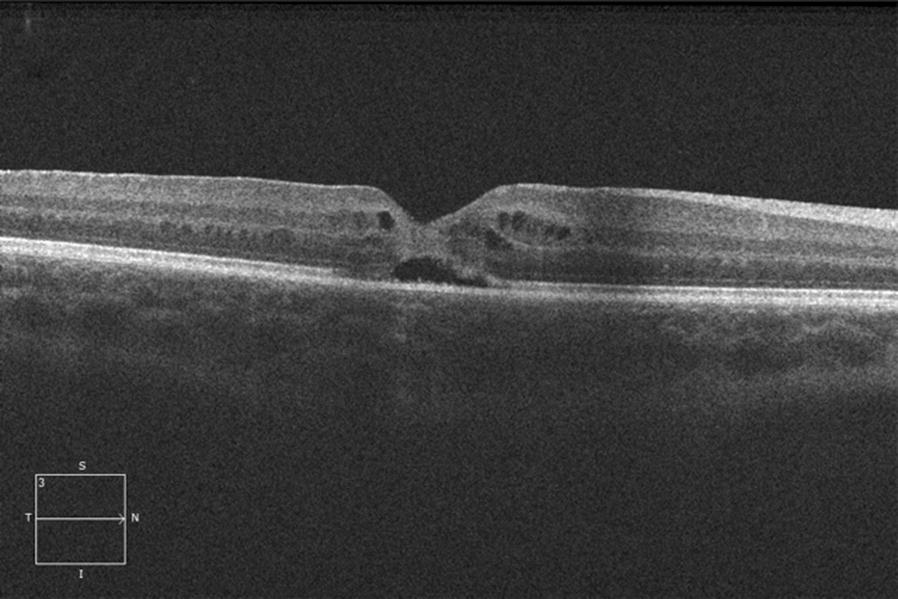
Horizontal high definition spectral domain OCT of the right eye 1 week post operatively shows closure of the macular hole

**Fig. 4:**
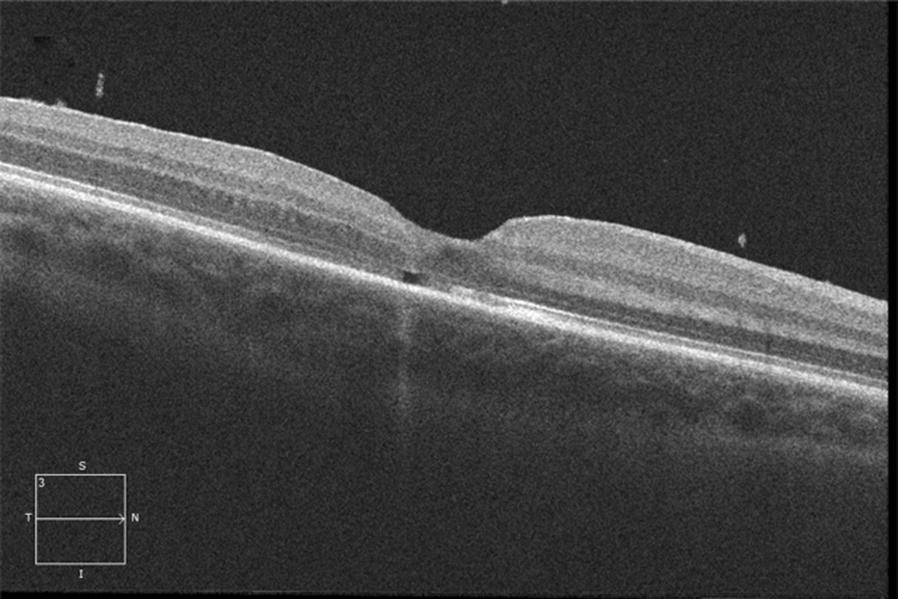
Horizontal high definition spectral domain OCT of the right eye 1 month post operatively shows further improvement in macular architecture

Following informed consent of her parents, we carried out a 23-gauge transconjunctival pars plana vitrectomy. Induction of a posterior vitreous detachment (PVD) was completed using triamcinolone, followed by ILM blue-assisted internal limiting membrane (ILM) peel, fluid–air exchange and air-26% sulfur hexafluoride (SF6) exchange. She was positioned face down for 3 days postoperatively. At postoperative week 1, VA was 20/60 with OCT evidence of hole closure (Fig. 3). At postoperative month 1, VA was 20/20, and at 4 months postoperative, the macular hole remained closed with remodeling of the outer retina on OCT (Fig. 4). There was still a small defect at the photoreceptor level.

## Discussion

There is only one previous report in the current literature of an idiopathic FTMH in a child. This case however, had features of cavitary maculopathy and did not have a full thickness defect [4].

In our case, the patient and her family were very reliable historians, strongly denying any possibility of antecedent trauma. Excellent vision of 20/17 was documented just 1 year prior by her optometrist. However, despite this, we must acknowledge, that given the active nature of children it is possible that an unreported trauma may have occurred in our case. Children often do not immediately report their recent behaviors or visual symptoms, possibly out of fear of retribution or simply a lack of understanding. In addition, the possibility of non-accidental injury should always be considered as other life-threatening injuries and or situations may also be present.

In the setting of blunt trauma, the most common cause of macular hole in children, a contrecoup mechanism as a result of axial globe compression is thought to increase vitreomacular traction forces [5]. Our case illustrates an idiopathic FTMH in a child, in which neither vitreomacular separation or vitreofoveal traction could be visualized clinically or on OCT. The mechanism of idiopathic FTMH formation in a child remains unclear.

Review of the English language literature identified 15 pediatric eyes with a non-traumatic macular hole (Table 1). In contrast to our case, all had clinical or historical features of a secondary non-traumatic cause including: vascular (retinopathy of prematurity (ROP) [6], and Coats’ disease [7, 8]), infective (Bartonella neuroretinitis [9–11]), and congenital (choroidal coloboma [12], regressed Bergmeister papilla [13]), and juvenile idiopathic epiretinal membrane [14]) entities. A case of accidental Nd:YAG laser induced macular hole in a child has also been reported, photothermal and photomechanical disruption of the retina occurring as a result of energy absorbed by the retinal pigment epithelium (RPE) [15]. The patients with ROP all had vitreoretinal surgery prior to discovery of the macular hole, and all had an associated retinal detachment. They were treated with a combination of open and closed vitrectomy in addition to a radial scleral buckle [6]. Of the 15 eyes, surgical hole closure was attempted in 9 cases. Apart from the patients with ROP, the other 5 cases were treated with vitrectomy, ILM peel and gas tamponade. Anatomic closure of the hole was reported in all 5 cases [4, 7, 13–15].

**Table 1:** Summary of reported non-traumatic full-thickness macular hole in paediatric patients

Author, case year	Associated cause of macular hole	No. of patients	Pt no.	Patient age (months, yrs), gender	Presenting BCVA	Size of hole (μm)	Surgery	Outcome	Last exam BCVA
Ahmad 2005	*Retinopathy of Prematurity*	5	1	10 months F	Not reported	Not reported	Yes Scleral buckle PPV + FAX	Large macular hole	20/3270
			2	3 years 2 months F	Not reported	Not reported	Yes PPV + ILM peel +FAX Cyanoacrylate glue applied to the hole Radial scleral buckle	Cyanoacrylate glue present Retina attached	20/760
			3	1 year 3 months F	Not reported	Not reported	Yes Radial sponge × 2	Retina attached	CF 2 feet
			4	1 year 2 months F	Not reported	Not reported	No	Total retinal detachment	NLP
			5	2 years M	Not reported	Not reported	Yes Radial sponge PPV + ILM peel + silicone oil	Retina attached	20/360
Albini 2005	*Bartonella Neuroretinitis*	1	1	10 years F	CF 1 foot	750 × 500 μm	No	Not reported	Not reported
Yokoyama 2005	*Juvenile Idiopathic Epiretinal Membrane*	1	1	3 years F	20/125	Not reported	Yes PPV + ILM Peel	Macular hole closed	20/80
Nakano 2005	*Incomplete regression of a Bergmeister Papilla*	1	1	10 years F	20/25	Not reported	Yes PPV + ICG ILM Peel + SF6 Post operative prone position 1 week	Macular hole closed	20/60
Donnio 2008	*Bartonella Neuroretinitis*	1	1	11 years M	20/200	Not reported	No	Not reported	Not reported
Kumar 2010	*Coats’ Disease*	1	1	9 years M	20/400	Not reported	No	Not reported	Not reported
Wong 2012	Coats’ Disease	1	1	10 years M	20/150	Not reported	Yes PPV + Autologous plasmin enzyme injection PPV + ICG ILM peel + C3F8 tamponade	Macular hole closed	20/60
Park 2012	*Idiopathic* *Cavitary Maculopathy*	1	1	8 years F	20/40	Not reported	Yes PPV + ILM peel + C3F8 Post operative prone position 2 weeks	Macular hole closed	20/40
Fernandez 2013	*Accidental Nd:YAG laser*	1	1	11 years M	20/100	1077 μm	Yes PPV + ILM Peel + C3F8 Post operative prone position 1 week	Macular hole closed	20/25
Seth 2015	*Bartonella Neuroretinitis*	1	1	11 years F	CF 1 foot	Not reported	No	Not reported	Not reported
Bansal 2017	*Choroidal Coloboma*	1	1	10 years F	20/60	Not reported	Not reported	Not reported	Not reported

*BCVA* best corrected visual acuity, *PPV* pars plana vitrectomy, *FAX* fluid air exchange, *ILM* internal limiting membrane, *CF* count fingers, *NLP* no light perception, *ICG* indocyanine green, *C3F8* Perfluoropropane, *SF6* sulfur hexafluoride

Macular surgery in the pediatric population has unique management and technical challenges. As previously mentioned, it may be difficult to accurately and reliably date how long the macular hole has been present. This poses a challenge when attempting to predict the presence of any contributory amblyopia to the presenting vision, and the potential visual benefit that can be expected from surgery. Induction of a PVD is difficult in children, and use of triamcinolone as in our case, may augment visualization of the posterior hyaloid. Fortunately for this patient, induction of the PVD was similar to that in an adult. Secondary complications including iatrogenic retinal tears and vitreous hemorrhage as a result of a young, adherent posterior hyaloid, in addition to late complications including vitrectomy induced cataract, should be considered carefully.

Finally, the ability to comply with post-operative care and practices including possible face down positioning are important in the pre-operative assessment of a child for macular surgery. Interestingly 3 cases specifically reported instructing their patient to position prone in the post-operative period [4, 13, 15]. These patients were of similar age to our patient, and also achieved macular hole closure.

## Conclusion

 This is the first reported case of an idiopathic FTMH in a child. Treatment with pars plana vitrectomy with peeling of the ILM resulted in significant anatomic and functional improvement.

## Abbreviations

FTMH: full thickness macula hole
VA: visual acuity
OCT: optical coherence tomography
PVD: posterior vitreous detachment
ILM: internal limiting membrane
SF6: sulfur hexafluoride
ROP: retinopathy of prematurity
RPE: retinal pigment epithelium
